# Incidentally Discovered Lown-Ganong-Levine Syndrome in a Patient Presenting With Acute Hypercapnic Respiratory Failure and Type II Myocardial Infarction: A Case Report

**DOI:** 10.7759/cureus.82087

**Published:** 2025-04-11

**Authors:** Saif M Srouji, Hamna Javed, Parisa Rezapoor, Mohammed G Elhassan

**Affiliations:** 1 Internal Medicine, Saint Agnes Medical Center, Fresno, USA

**Keywords:** acute hypoxemic respiratory failure, cardio vascular disease, ecg abnormalities, internal medicine, invasive mechanical ventilation, lown-ganong-levine syndrome, non-st segment elevation acute coronary syndrome, pulmonary critical care, short pr interval, unusual cause of syncope

## Abstract

Lown-Ganong-Levine (LGL) syndrome is a rare pre-excitation disorder associated with paroxysmal tachyarrhythmias. We present a case of a 53-year-old male with no significant medical history who was found unconscious in his car with dry ice exposure. He was tachypneic, hypotensive, and encephalopathic, requiring intubation for acute hypercapnic respiratory failure. Investigations revealed non-ST elevation myocardial infarction (NSTEMI) that was thought to be secondary to increased myocardial demand in the setting of respiratory failure, acute kidney injury (AKI), and nephrotic-range proteinuria. Electrocardiography (ECG) showed short PR intervals consistent with LGL syndrome. He was not known to have any prior history of arrhythmias. Coronary angiography did not show any obstructive coronary artery disease (CAD). The patient improved, was started on a beta-blocker as a preventative measure to reduce the risk of development of tachyarrhythmias, and discharged with cardiology follow-up on an outpatient basis to complete outpatient cardiac monitoring and assess the need for a cardiac electrophysiology study. This case highlights the diagnostic challenges of LGL syndrome coexisting with critical illness.

## Introduction

Lown-Ganong-Levine (LGL) syndrome is a rare pre-excitation disorder characterized by a short PR interval (<120 ms), a normal QRS complex, and paroxysmal tachyarrhythmias due to the potential presence of an accessory pathway that bypasses the atrioventricular (AV) node without causing ventricular pre-excitation, often referred to as the Bundle of James [1]. However, the implication of accessory pathways in this condition remains debatable. Unlike Wolff-Parkinson-White (WPW) syndrome, LGL syndrome lacks delta waves on ECG, making its diagnosis more challenging [1]. While LGL syndrome is often thought to be associated with supraventricular tachyarrhythmias as in WPW, its link with acute coronary syndromes, including non-ST elevation myocardial infarction (NSTEMI), is extremely rare and poorly understood.

Syncope in LGL syndrome is typically attributed to rapid atrial arrhythmias leading to hemodynamic instability, but in some cases, it may indicate underlying ischemic heart disease [2]. The coexistence of LGL syndrome with ACS raises important clinical considerations, as tachyarrhythmias can increase myocardial oxygen demand and contribute to ischemic events, while coronary artery disease (CAD) may exacerbate arrhythmogenic potential [3].

Here, we present a rare case of a patient with incidentally discovered LGL syndrome who presented due to acute respiratory failure and NSTEMI, emphasizing the diagnostic challenges and therapeutic implications of managing both arrhythmias and possible ischemic heart disease in such patients, as investigators and treatment approaches will vary widely between LGL syndrome, as opposed to other causes of supraventriuclar tachycardia such as physiological sinus tachycardia.

## Case presentation

A 53-year-old male with no significant past medical history presented to the emergency department (ED) via ambulance after being found unconscious in his car, foaming at the mouth and with pinpoint pupils. It was noted that the vehicle was running, with the windows closed and the doors locked. En route to the ED, his blood glucose level was found to be 275 mg/dL, and he was given a total of 4 mg of naloxone empirically, which did not appear to improve his condition.

Upon initial assessment in the ED, the patient was noted to be tachypneic, with a respiratory rate (RR) of 24 breaths per minute, and slightly hypotensive, with a blood pressure (BP) of 99/71 mmHg. Foamy secretions were noted in his mouth, and he exhibited labored breathing. He was moaning, moving all extremities, and opening his eyes in response to painful stimuli. Emergency medical service (EMS) personnel noted that the patient had nearly 200 pounds of dry ice (solid CO₂) in the vehicle, which was initially considered the most likely cause of his presentation. An urgent venous blood gas (VBG) revealed hypercapnic respiratory acidosis, with values presented in Table 1.

**Table 1:** Peripheral venous blood gas (VBG) results. A peripheral venous blood gas obtained urgently on initial presentation indicating hypercapnic respiratory acidosis.

Parameters	Patient values	Lab-provided reference range
pH	7.04	7.31-7.41
pCO_2_	104.9 mmHg	41-51 mmHg
pO_2_	23 mmHg	80-105 mmHg
HCO_3_	28.7 mmol/L	22-28 mmol/L
Base excess	-2 mmol/L	-2 to 3 mmol/L

He remained encephalopathic and was not protecting his airway, necessitating intubation and the initiation of mechanical ventilation. Fentanyl and propofol infusions were started for sedation. Other laboratory results were notable for the values presented in Table 2.

**Table 2:** Notable laboratory investigation values obtained on admission.

Parameters	Patient values	Reference range
Creatinine	1.46 mg/dL	0.5-1.2 mg/dL
Estimated glomerular filtration rate (eGFR)	58 mL/minute/1.72 m^2^	60-999 mL/minute/1.72 m^2^
White blood cells (WBC)	18 K/mcL	4.5-11 K/mcL
Ammonia	55 mcmol/L	11-35 mcmol/L
High-sensitivity (HS) troponin	576 ng/L	0-20 ng/L
Acetaminophen level	<10 mcg/mL	10-25 mcg/mL
Salicylate level	<2.5 mg/dL	0-30 mg/dL
Ethanol level	<10 mg/dL	0-10 mg/dL
TSH	0.43 mcIU/mL	0.45-5.33 mcIU/mL
Free T4	0.62 ng/dL	0.58-1.64 ng/dL
Urine protein level	>500 mg/dL	Negative
Urine glucose level	150 mg/dL	Negative
Urine drug screen	+ Cannabinoid Screen	High sensitivity

**Figure 1:**
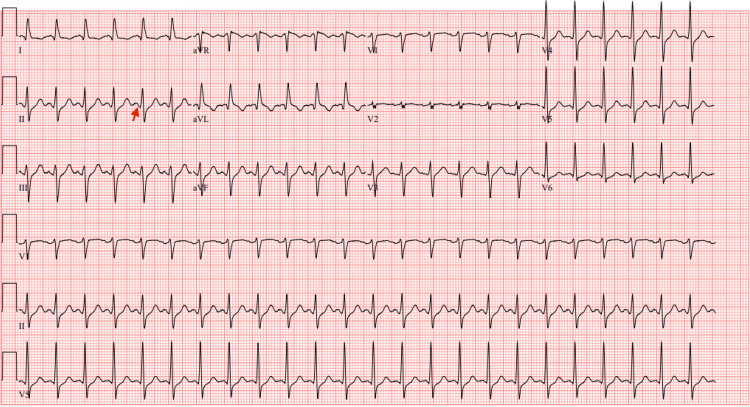
An electrocardiogram (ECG) showing a shortened PR interval ECG showing sinus tachycardia with a heart rate (HR) of 145 beats per minute. The PR interval (red arrow) appears visibly shortened and was measured at 116 ms (ref. range 120-200 ms), in addition to the left anterior fascicular block. No acute ischemic changes were seen in this ECG in light of the elevated troponin, so this was labeled as non-ST elevation myocardial infarction (NSTEMI).

An ECG revealed sinus tachycardia with a heart rate (HR) of 145 beats per minute without any acute ischemic changes. The PR interval was measured at 116 ms, which is slightly below the normal reference range of 120-200 ms (Figure 1). A chest X-ray showed no acute cardiopulmonary abnormalities. A CT scan of the head was also obtained and revealed no acute intracranial abnormalities.

The critical care team was consulted for admission, and the patient was transferred to the intensive care unit (ICU) with acute hypoxic and hypercapnic respiratory failure and acute metabolic encephalopathy. He was also developing a myocardial infarction (MI), likely due to increased oxygen demand; therefore, a heparin drip was not initiated initially. Additionally, he was found to have acute kidney injury associated with proteinuria. The Cardiology team was consulted to evaluate the elevated troponin levels.

**Figure 2:**
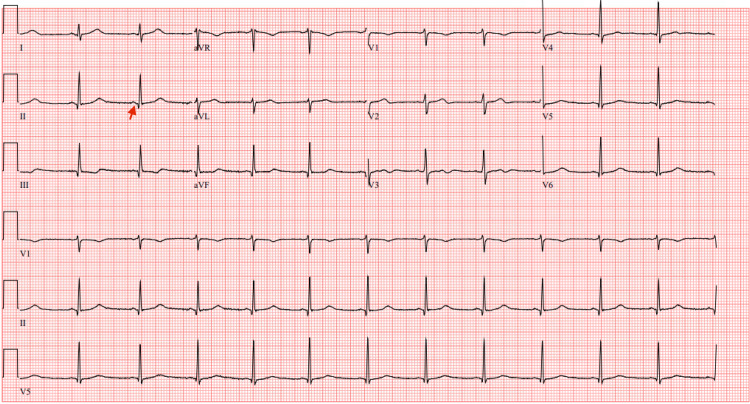
An electrocardiogram (ECG) showing a shortened PR interval. A repeat ECG showing narrow QRS complexes of 80 ms duration (reference range 80-120 ms) and short PR intervals (red arrow) of 103 ms duration (reference range 120-200 ms) consistent with Lown-Ganong-Levine (LGL) syndrome.

The following morning, sedation was weaned, and the patient was no longer encephalopathic. He passed his spontaneous awakening trial (SAT) with a Glasgow Coma Score (GCS) of 15. He was able to communicate with the medical team using pen and paper. A spontaneous breathing trial (SBT) was performed concurrently and was successful; therefore, the patient was extubated to a nasal cannula. Upon further discussion with him about the events of the previous day, he denied using drugs or alcohol. He mentioned that he did not remember much beyond being in his car. He did recall feeling suddenly short of breath shortly before losing consciousness but denied experiencing any chest pain or palpitations. He also mentioned that he had no prior history of arrhythmias and does not typically experience palpitations. At this time, a repeat ECG showed narrow QRS complexes with a duration of 80 ms (reference range: 80-120 ms) and short PR intervals measuring 103 ms (reference range: 120-200 ms), findings consistent with LGL syndrome (Figure 2).

He was asymptomatic at this time. Daily aspirin 81 mg orally and atorvastatin 20 mg were initiated. A transthoracic echocardiogram (TTE) was performed, which was normal with no wall motion abnormalities, except for a left ventricular ejection fraction (LVEF) of 50%-55%. A repeat troponin level was 2,977 ng/mL before it began to decline. Following a discussion with the cardiology team, an unfractionated heparin infusion was initiated, and the patient was scheduled for coronary angiography the following day. He was deemed to be in stable condition and was subsequently transferred to the medical floor. A renal ultrasound was unremarkable, showing only medical renal disease. Repeated kidney function tests and urinalysis had returned to normal ranges, suggesting that the acute kidney injury (AKI) was likely secondary to the acute illness.

**Figure 3:**
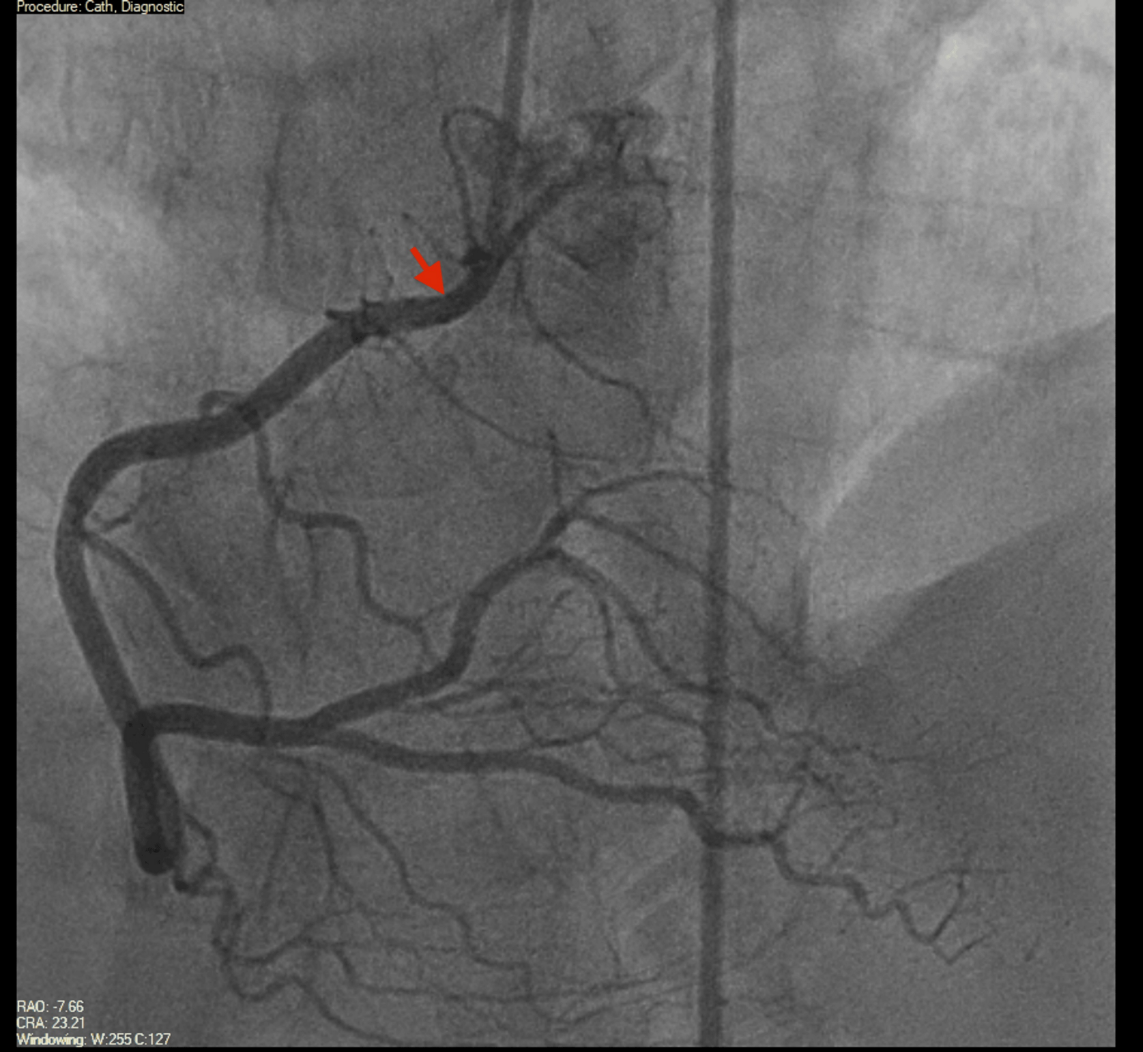
Coronary angiography showing a normal, large-caliber right coronary artery (RCA) (red arrow) without significant coronary artery disease (CAD).

**Figure 4:**
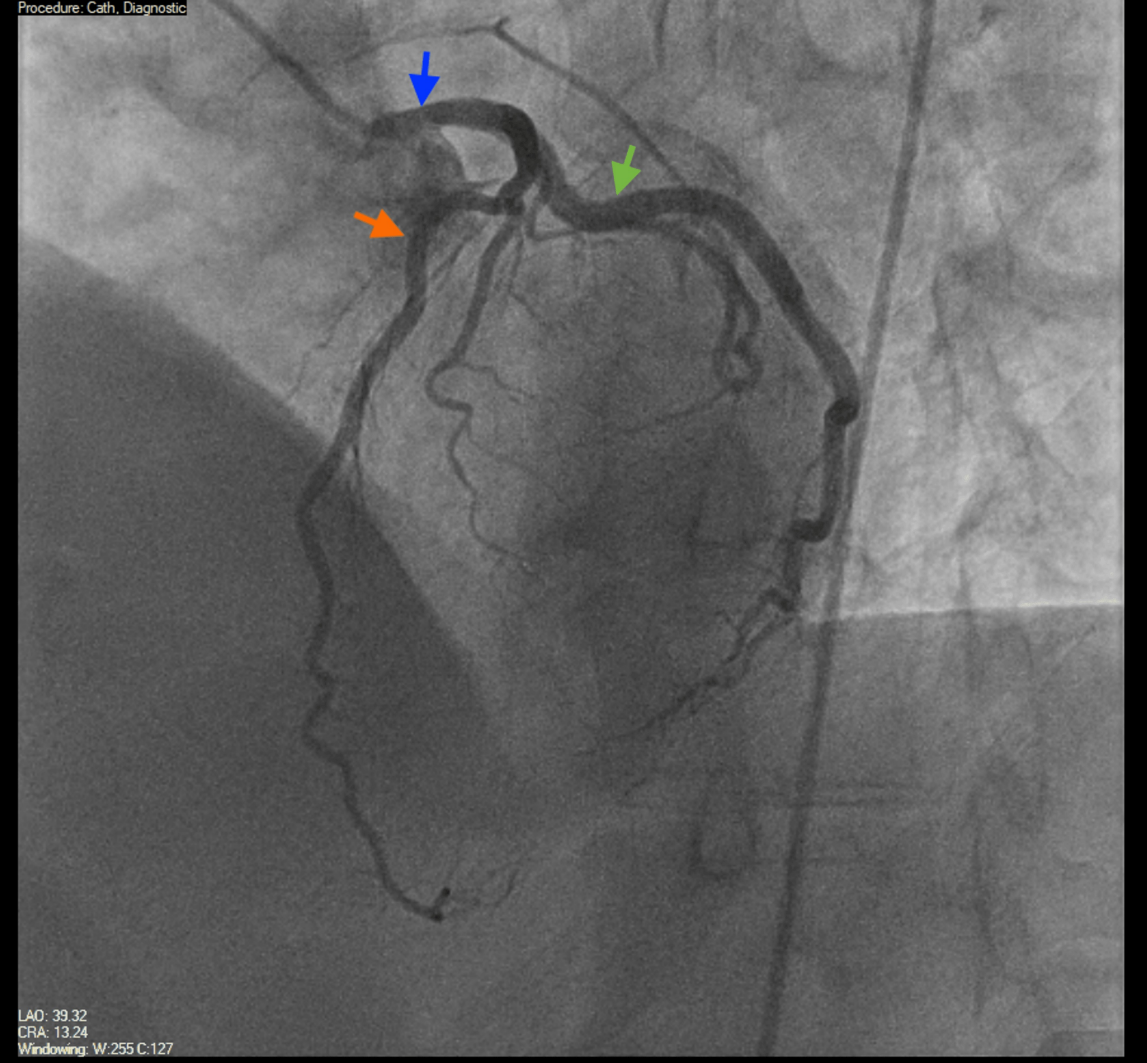
Coronary angiography visualizing the left main coronary artery (LMA) (blue arrow), left anterior descending artery (LAD) (orange arrow), and left circumflex artery (LCX) (green arrow), all without significant coronary artery disease.

**Figure 5:**
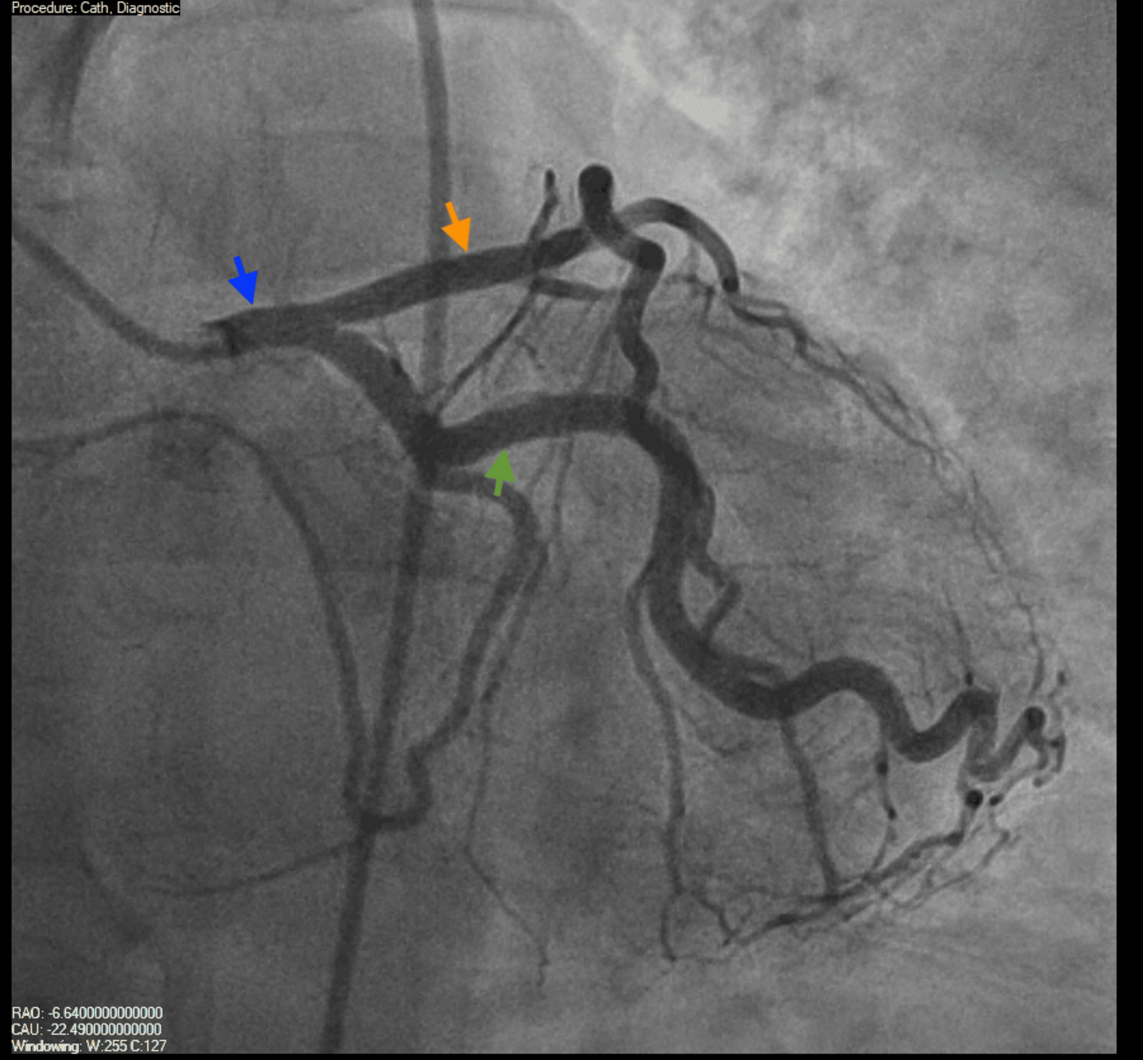
Coronary angiography visualizing the left main coronary artery (LMA) (blue arrow), left anterior descending artery (LAD) (orange arrow), and left circumflex artery (LCX) (green arrow). All vessels are without significant coronary artery disease.

**Figure 6:**
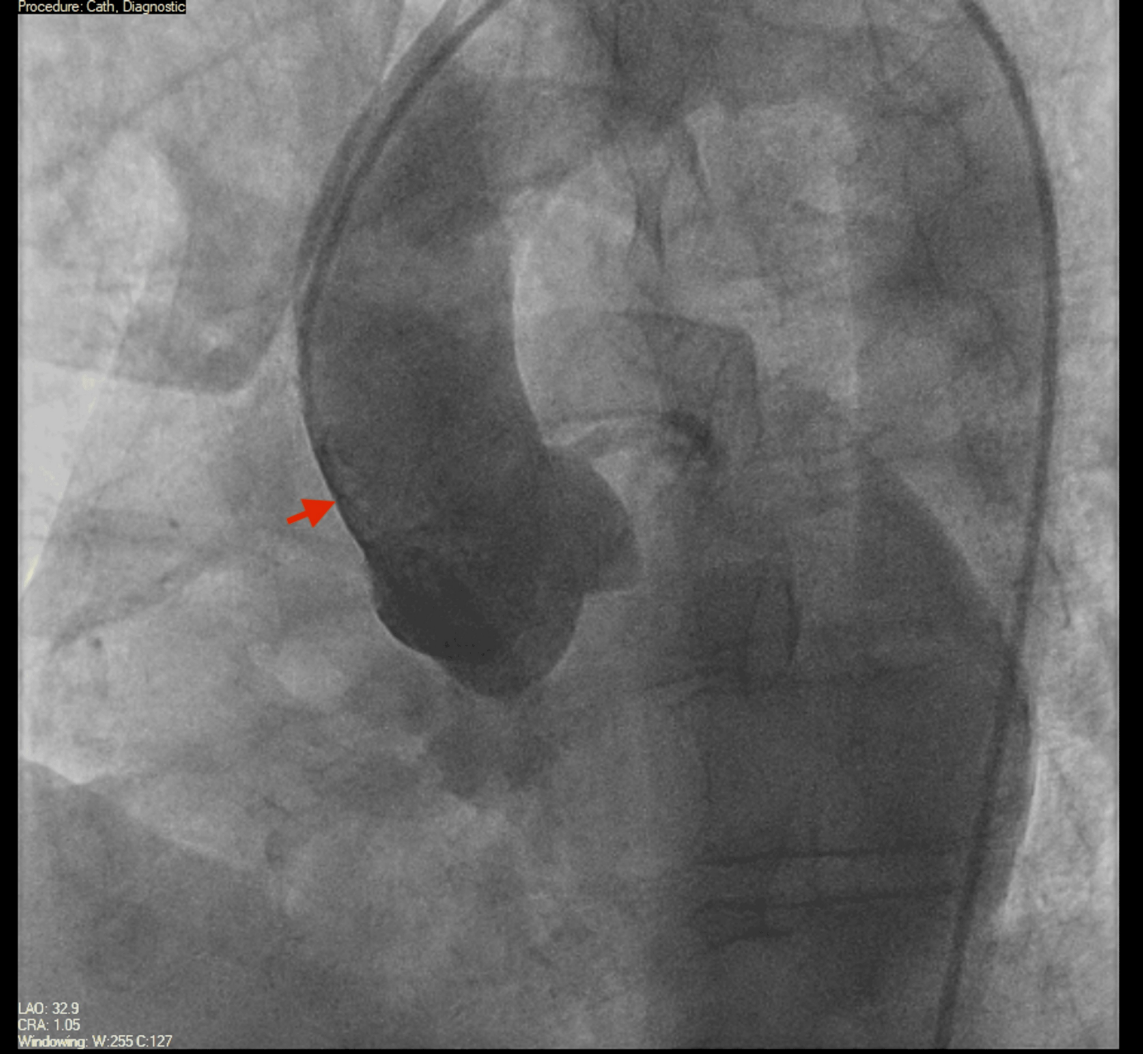
Aortography showing a normal ascending aorta. Aortography performed during left heart catheterization visualizing the ascending aorta (red arrow) that looks normal.

On day 3 of admission, he underwent coronary angiography, which showed large-caliber coronary vessels without obstructive lesions (Figures 3-5). The aortic root was also examined and was found to be normal (Figure 6). The Heparin infusion was stopped after completing 48 hours of treatment.

He was later discharged home without any major complications and was advised to take metoprolol succinate 25 mg p.o. daily. A follow-up with cardiology was scheduled for the initiation of ambulatory cardiac monitoring and evaluation of the need for a cardiac electrophysiology study.

## Discussion

The LGL syndrome is an electrocardiographic finding that is a pattern best described by the presence of a short PR interval of less than 120 ms (reference range 120-200 ms) with a normal QRS complex. It was first described in 1938 as an ECG finding in patients who were complaining of palpitations, and it was further studied by Lown, Ganong, and Levine in 1952, hence the name. This study aimed to clarify the relationship of short PR interval ECG findings to WPW syndrome and concluded that there was an entity that resembled WPW syndrome but was visibly distinct; this entity was later named LGL syndrome [1].

This ECG phenomenon is traditionally thought to be associated with either an accessory pathway that bypasses the atrioventricular (AV) node or with enhanced conduction through the AV node itself. This conduction pathway, often referred to as the bundle of James, electrically connects the atrial myocardium directly to the bundle of His. Because this abnormal pathway bypasses the normal conduction system, it results in a shortened PR interval on the ECG, which is a direct consequence of the electrical impulse conducting more rapidly than usual. However, the subsequent QRS complex appears normal as the impulse continues through the usual pathway via the bundle of His, leading to normal ventricular depolarization [4]. However, it is worth mentioning that studies have often failed to prove the presence of a distinct pathway in several patients [5]. This discrepancy in theory and evidence can be considered a limiting factor in the understanding and interpretation of the clinical importance of this syndrome.

The main electrocardiographic difference between LGL and WPW syndromes is that LGL lacks the *delta wave* seen in WPW. Physiologically, the accessory pathway in WPW connects the myocardial muscle to the ventricular muscle, bypassing the bundle of His, which results in the presence of *delta waves* [6], while in LGL syndrome, the connection is to the bundle of His; hence, *delta waves* are not present. Making this distinction is important, as the rates of complications differ between the syndromes, and recognizing this difference may help inform clinical judgment during management decisions.

The prevalence of LGL syndrome is reported to be less than 1 per 1 million [7]. Recognition of this electrocardiographic pattern is important because it has been potentially linked to the development of paroxysmal supraventricular tachycardia (SVT), which can theoretically lead to dangerous arrhythmias and subsequently further complications. In LGL specifically, this association is thought to be due to orthodromic AV reentry tachycardia (AVRT) using an accessory pathway [8,9]. As this syndrome is rare and large studies regarding outcomes are lacking, it is hard to accurately estimate the incidence of serious life-threatening or clinically significant tachyarrhythmias. However, in their original article, Lown et al. reported the incidence of paroxysmal tachycardia in 10.4% of the LGL syndrome group as opposed to 25% in the WPW syndrome group [1]. Due to the aforementioned reasons, this is recognized as a major limitation. Data of this nature should be interpreted with caution, especially when used to guide clinical decisions, as a clear mechanism linking LGL syndrome to SVT remains unclear.

In light of this uncertainty regarding the clinical significance of this syndrome, no clear management guidelines exist, and specialists often use clinical conditions to guide management instead of relying solely on the presence of the syndrome. It is reasonable to consider two long-term approaches for treatment based on general management guidelines for SVT, a medical approach and a procedural approach. The medical approach would be through the utilization of oral agents like B-blockers, calcium channel blockers (Verapamil, Diltiazem), and Digoxin, which exert an effect through slowing conduction via the AV node; however, these agents may be contraindicated in cases where an accessory pathway exists. The use of Classes I and III antiarrhythmic medications (Flecainide, Amiodarone, Sotalol) in these cases may also be reasonable as they may slow down conduction through accessory pathways. The procedural approach typically consists of radiofrequency ablation for accessory pathways in symptomatic patients with an evident pathway. In cases where life-threatening ventricular tachyarrhythmias occur as a complication, it may also be reasonable to use an approach based on AV nodal ablation and permanent pacemaker implantation [10]. Thus, to guide medical treatment, it is important to confirm the presence or absence of accessory pathways via a cardiac electrophysiology study.

Some studies have reported an association between SVTs in general and elevations in serum troponin without the presence of severe CAD; however, further decisions in the management plan should rely on an objective assessment of the individual's risk factors as assessed by the clinician [11,12]. In our case, the theory that the patient may have experienced a paroxysm of SVT leading to loss of consciousness or increased myocardial oxygen demand, and consequently a type II MI, remains speculative. The fact that dry ice (solid CO₂) was also implicated in this presentation and led to acute hypercapnic respiratory failure is a more likely scenario, especially given the presence of objective evidence.

## Conclusions

This case highlights the coexistence of acute encephalopathy and demand ischemia in a patient with LGL syndrome, emphasizing the complexities of diagnosing and managing arrhythmia-related cardiovascular events, particularly in the presence of multiple potential confounders. While LGL syndrome is typically associated with supraventricular tachyarrhythmias, its role in directly precipitating ischemic events remains unclear. However, it may be explained by increased myocardial demand as a complication of tachyarrhythmias. The absence of obstructive CAD suggests that demand ischemia secondary to tachyarrhythmia may have contributed to the MI, although respiratory failure is a more realistic scenario. Early recognition, close cardiac monitoring, and appropriate medical therapy, including beta-blockers, are essential in such cases, except where accessory pathways are present, as this is usually contraindicated. While LGL syndrome remains a poorly established diagnosis, further studies are needed to understand the pathophysiologic mechanisms linking LGL syndrome, or tachyarrhythmias in general, to myocardial ischemia.
